# Taxon Richness of “Megaviridae” Exceeds those of Bacteria and Archaea in the Ocean

**DOI:** 10.1264/jsme2.ME17203

**Published:** 2018-05-25

**Authors:** Tomoko Mihara, Hitoshi Koyano, Pascal Hingamp, Nigel Grimsley, Susumu Goto, Hiroyuki Ogata

**Affiliations:** 1 Bioinformatics Center, Institute for Chemical Research, Kyoto University Uji, Kyoto 611–0011 Japan; 2 School of Life Science and Technology, Laboratory of Genome Informatics, Tokyo Institute of Technology 2–12–1 Ookayama, Meguro-ku, Tokyo 152–8550 Japan; 3 Aix Marseille Université, Université de Toulon CNRS, IRD, MIO UM 110, 13288, Marseille France; 4 Integrative Marine Biology Laboratory (BIOM), CNRS UMR7232, Sorbonne Universities 66650, Banyuls-sur-Mer France; 5 Database Center for Life Science, Joint-Support Center for Data Science Research, Research Organization of Information and Systems Wakashiba, Kashiwa, Chiba 277–0871 Japan

**Keywords:** *Mimiviridae*, Megaviridae, species richness, RNA polymerase, ocean metagenome

## Abstract

Since the discovery of the giant mimivirus, evolutionarily related viruses have been isolated or identified from various environments. Phylogenetic analyses of this group of viruses, tentatively referred to as the family “Megaviridae”, suggest that it has an ancient origin that may predate the emergence of major eukaryotic lineages. Environmental genomics has since revealed that Megaviridae represents one of the most abundant and diverse groups of viruses in the ocean. In the present study, we compared the taxon richness and phylogenetic diversity of Megaviridae, Bacteria, and Archaea using DNA-dependent RNA polymerase as a common marker gene. By leveraging existing microbial metagenomic data, we found higher richness and phylogenetic diversity in this single viral family than in the two prokaryotic domains. We also obtained results showing that the evolutionary rate alone cannot account for the observed high diversity of Megaviridae lineages. These results suggest that the Megaviridae family has a deep co-evolutionary history with diverse marine protists since the early “Big-Bang” radiation of the eukaryotic tree of life.

*Acanthamoeba polyphaga mimivirus* (APMV), initially mistaken as a Gram-positive bacterium when it was isolated via amoeba co-culture in the 1990s (4), was recognized in 2003 as a *bone fide* virus, indeed a ‘giant virus’ with a large 750-nm virion including a fibril-containing rigid surface layer (40). APMV possesses a 1.2-Mb linear dsDNA genome coding for more than 1,000 genes (43, 65), which is more than those encoded on the genomes of some small prokaryotes. It is classified as a member of nucleocytoplasmic large DNA viruses (NCLDVs), the proposed order “Megavirales” (17), together with various giant viruses discovered after APMV (6, 45, 46, 63, 78). The unexpected dimensions and complexity of APMV and other exotic giant viruses triggered the reassessment of differences between cellular and viral life forms (66), fueled debates on the origin of viruses (14, 54, 56), and revived interest in re-defining the concept of viruses (13, 15, 27).

Phylogenetic studies have indicated multiple origins of APMV genes; some APMV genes appear to be of viral origin, whereas others appear to originate from cellular organisms (23, 24, 55, 71) or unknown sources (*i.e.*, ORFans). Despite the apparent mosaicity of its complex genome, one coherent finding that emerges from these studies is that the origin of APMV lineage is old, being as ancient as the emergence of the Eukarya domain in the Tree of Life (65). In particular, the ancient origin of APMV and related giant viruses has been supported by phylogenies of replication- and transcription-related genes (1, 72, 77, 89). The antiquity of giant viruses further inspired hypotheses of a putative “Fourth Domain of Life”, although these are still highly controversial (7, 16, 44, 57, 59, 83, 90).

Since the discovery of APMV, numerous APMV relatives have been isolated using amoeba co-culture from different environments including marine sediment, river, soil, contact lens liquid, and sewage water (2, 31, 41, 88). These viruses are subdivided into lineage A, B and C mimiviruses (88). These amoebal mimiviruses, together with additional giant viruses infecting microzooplankton, such as *Cafeteria roenbergensis virus* (CroV) (25) and *Bodo saltans virus* (19) as well as Klosneuviruses recently identified in metagenomes (71), constitute the family *Mimiviridae* officially approved by the International Committee on Taxonomy of Viruses (ICTV). Shortly after the discovery of APMV, algal viruses isolated in the sea, such as *Chrysochromulina ericina virus* (CeV) and *Pyramimonas orientalis virus*, were found to form a strongly supported monophyletic group with APMV based on DNA polymerase phylogenies (28, 51, 52). Since then, the monophyletic group has grown with the inclusion of *Phaeocystis globosa virus* (PgV) (69), *Aureococcus anophagefferens virus* (AaV) (53), *Haptolina ericina virus* (HeV RF02), and *Prymnesium kappa viruses* (PkV RF01 and PkV RF02) (34) as well as metagenome-assembled Organic lake phycodnaviruses (OLPV1 and OLPV2) (86) and Yellowstone lake mimivirus (YSLGV) (91). Some of these viruses are officially, but inappropriately, classified in the *Phycodnaviridae* family. Arslan *et al.* proposed to reassign the family “Megaviridae” to the monophyletic group that combines the above mentioned mimiviruses, zooplankton giant viruses, and algal giant viruses (2). Gallot-Lavallée *et al.* recently proposed to classify mimiviruses and microzooplankton giant viruses of the Megaviridae family into the subfamily “Megamimivirinae” and the algal viruses into the subfamily “Mesomimivirinae” (29). The tentative Megaviridae family is the focus of the present study and it is this proposed Megaviridae nomenclature that we use henceforth.

Megaviridae constitutes approximately 36% of giant viruses in epipelagic oceans, with their abundance being in the order of 10^3^ to 10^5^ genomes mL^−1^ sea water (32). A recent metatranscriptomic study also demonstrated that members of Megaviridae are active everywhere in sunlit oceans and infect eukaryotic communities of various size ranges from piconano-plankton (0.8–5 μm) up to mesoplankton (180–2,000 μm) (9). The hosts of isolated Megaviridae are still limited to a handful of eukaryotic lineages, but already encompass an extremely wide range of unicellular eukaryotes, including Amoebozoa, Stramenopiles (Cafeteriaceae and Pelagophyceae), Euglenozoa (Kinetoplastida), Haptophyceae (Phaeocystales and Prymnesiales), and Viridiplantae (Chlorophyta). Sequence similarity searches between metagenomic sequences and known Megaviridae genomes also indicated the existence of many uncultured Megaviridae lineages in marine environments (32). Taken together with the inferred antiquity of Megaviridae, these findings suggest that the host range of Megaviridae is markedly wider than currently recognized, and species richness inside the family Megaviridae may consequently be comparable with that of protists, which undoubtedly represent the major part of eukaryotic species’ diversity.

DNA-dependent RNA polymerases (RNAPs) of cellular organisms are multisubunit protein complexes, the structures of which have been elucidated for all three domains of life (Bacteria, Archaea, and Eukarya) (82). The number of subunits constituting the machinery differs across the domains of life (50, 82). Among them, the two largest subunits (hereafter referred to as Rpb1 and Rpb2) are both highly conserved and mostly encoded as single copy genes in the three domains of life, although eukaryotes commonly possess distant paralogs (33). Rpb1 and Rpb2 of eukaryotes correspond to the RNA polymerase β’ and β subunits of bacteria, and to the RpoA and RpoB of archaea, respectively (39, 80, 81). Archaeal RpoA is composed of two subunits encoded by two small genes. Rpb1 and Rpb2 have been selected as two of the 102 genes suitable for the assessment of phylogenetic relationships among prokaryotes (*i.e.*, 102 Nearly Universal Trees) (38). Moreover, Rpb1 and Rpb2 are conserved in all known members of Megaviridae (50). Transcriptomic and proteomic studies have indicated that Rpb1 and Rpb2 are expressed during infection and packed into mimivirus capsids (12, 42, 43, 67). Bacteriophages, such as T7 and SP6, encode single-subunit RNAPs, which are phylogenetically unrelated to multisubunit RNAPs (10, 74). Therefore, Rpb1 and Rpb2 possess the required characteristics to be used as phylogenetic markers for both Megaviridae and cellular organisms (65, 72). In the present study, we investigate the taxon richness (or lineage richness) and phylogenetic diversity (PD) of Bacteria, Archaea, and Megaviridae based on Rpb1 and Rpb2 sequences found in marine microbial metagenomes derived from prokaryotic size fractions.

## Materials and Methods

### Sequence data

We used the UniProt database (Release 2016_03) (79) and the National Center for Biotechnology Information (NCBI) Reference Sequence (RefSeq) (61) Viral Section (Release 75) database to collect Rpb1 and Rpb2 protein sequences from cellular organisms and NCLDVs. We additionally used the GenomeNet/Virus-Host Database (49) to retrieve the nucleotide sequences of Rpb1 and Rpb2.

Marine metagenomic sequence data were obtained from CAMERA (75) and the *Tara* Oceans project (32, 76) (Table S1). In addition, we obtained metagenomic data for other non-marine environments from CAMERA and KEGG/MGENES (35). Collectively, we used metagenomic data derived from 58 projects (Table S1). Based on metagenomic data, we initially prepared files for amino acid sequences for open reading frames (ORFs) that were longer than or equal to 150 codons. The total number of ORFs was 149,645,996: marine metagenomes (101,856,227 ORFs, 68%), other aquatic environmental metagenomes (8,385,210 ORFs, 5.6%), mammal-associated microbial metagenomes (38,341,510 ORFs, 26%), and other metagenomes (1,063,049 ORFs, 0.7%). Most of the analyses presented in the present study focused on data from marine metagenomes, mainly derived from two large scale oceanic microbiome projects: *Tara* Oceans and the Global Ocean Sampling project (68). Data from other environments were used to confirm marine data.

### Non-synonymous and synonymous substitution rate ratio

In order to estimate the level of functional constraint on Rpb1/Rpb2 coding sequences, we computed the numbers of non-synonymous (Ka) and synonymous (Ks) substitutions per site and their ratio (ω=Ka/Ks) using a maximum likelihood method implemented in the codeml program in the PAML package (85). We used the Mann-Whitney U test to assess the significance of differences in ω values between Megaviridae and bacterial sequences.

### Reference sequence alignments and phylogenetic trees

We identified Rpb1 and Rpb2 homologs in the UniProt and RefSeq databases using HMMER/HMMSEARCH (version 3.1; E-value<1×10^−5^) (20) based on profile hidden Markov models that we built from alignments of Rpb1 (COG0086) and Rpb2 homologs (COG0085) (30). We used CD-HIT version 4.6 to reduce the redundancy of the collected known Rpb1/2 sequences (47). The resulting non-redundant sequences were aligned using MAFFT v7.215 (36) with default parameters and alignment columns containing gaps were trimmed using trimAl v1.2rev59 (8). We referred to the resulting reference sequence alignments for Rpb1 and Rpb2 as RAln-Rpb1 and RAln-Rpb2, respectively. We also generated reference sequence alignments solely composed of sequences from Megaviridae, Bacteria, and Archaea, and referred to the alignments as RAln-MBA-Rpb1 and RAln-MBA-Rpb2. Maximum likelihood phylogenetic trees were constructed with the use of FastTree version 2.1.7 (64). The resulting reference trees for Rpb1 and Rpb2 were referred to as RTree-Rpb1 and RTree-Rpb2, respectively. The significance of the branches in the trees was assessed using the Shimodaira-Hasegawa test (73) implemented in FastTree. Reference alignments and trees are available at the GenomeNet ftp site (ftp://ftp.genome.jp/pub/db/community/RNAP_ref_tree).

### Identification of RNAP homologs in metagenomes

In order to identify Rpb1 and Rpb2 homologs in metagenomic sequence data, we used HMMER/HMMSEARCH (version 3.1) (20) with the default parameters and built 10 HMMs for Rpb1 and 10 HMMs for Rpb2, each of which represents a group of phylogenetically related sequences in our reference phylogenetic trees. Specifically, these HMMs represent Megaviridae, other NCLDV groups 1 and 2 (group 1: *Asfarviridae*, *Poxviridae*; 2: *Ascoviridae*, *Iridoviridae*, Pandoravirus, Pithovirus), Bacteria, Archaea, Eukaryotes I to IV (I: RNAP I; II: RNAP II; III: RNAP III; IV: RNAP IV/V), and RNA polymerases of plastids. We screened metagenomic data for the Rpb1 and Rpb2 homologs (≥150 amino acid residues) using these profile HMMs with HMMSEARCH (E-value<1×10^−5^).

### Taxonomic classification

Phylogenetic placement is a bioinformatics technique that is used to identify the most likely phylogenetic position for a given query sequence on a reference phylogenetic tree. Pplacer is one of the phylogenetic placement tools that efficiently analyze large numbers of sequences, including short metagenomic sequences, within linear computation time (48). Metagenomic Rpb1/Rpb2 sequence fragments were aligned on the reference alignments (*i.e.*, RAln-Rpb1 and RAln-Rpb2) using HMMALIGN and placed on the reference phylogenetic trees (RTree-Rpb1 and RTree-Rpb2) using Pplacer with the use of the maximum likelihood mode. These Rpb1 and Rpb2 fragments were taxonomically classified into the above-mentioned 10 phylogenetic groups based on their phylogenetic placement.

### Taxon richness and PD

Metagenomic Rpb1/Rpb2 fragments that were taxonomically assigned to Megaviridae, Bacteria, or Archaea were re-aligned on the RAln-MBA-Rpb1 or RAln-MBA-Rpb2 reference sequence alignments using HMMALIGN. Since metagenomic sequences were often shorter than full-length sequences in the reference alignments, we examined taxon richness (*i.e.*, the number of sequence clusters) (3) and PD (22) along the alignment using a 100-residue sliding window on the alignments (with a step size of 10 residues). Metagenomic sequences exhibiting gaps at >10% of the sites in the alignment window were discarded.

Taxon richness was computed based on sequence clustering by the ucluster_fast command of USEARCH v7.0 (21) with three cutoff values for amino acid sequence identities (*i.e.*, 70%, 80%, and 90%). The significance of differences between richness curves was assessed using a Log-rank test (70).

PD was calculated using Phylogenetic Diversity Analyzer (PDA) version 1.0 (11), based on FastTree phylogenetic trees of metagenomic sequences that were aligned inside the sliding window. In order for PD scores to be comparable between Megaviridae, Bacteria, and Archaea, we constructed a phylogenetic tree with 1,000 randomly selected sequences for each organism group and calculated the PD score.

### RNAP paralogs in Megaviridae

Some of the sequenced viruses of Megaviridae, such as PgV (69), OLPV1, and OLPV2, encode two distantly related Rpb2 in their genomes. In order to eliminate the effect of the presence of these paralogs on the richness assessment of Megaviridae Rpb2, we classified Megaviridae Rpb2 metagenomic sequences into two paralogous groups based on phylogenetic reconstructions and performed additional rarefaction analyses for each of the paralogous groups.

## Results

### Functional constraints on Megaviridae Rpb2 and Rpb1 are higher than those on bacterial homologs

**Fig. 1:**
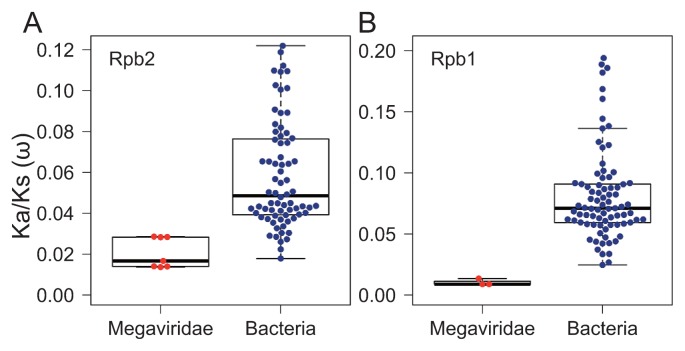
Functional constraints on Megaviridae Rpb2 and Rpb1. Non-synonymous (Ka) and synonymous (Ks) substitution rate ratios (ω=Ka/Ks) are plotted for Megaviridae and bacterial Rpb2 (A) and Rpb1 (B). We selected pairs of orthologs from Megaviridae (shown in red dots) based on the following criteria: Ka>0.01, Ks<5.0, and the percent standard error of ω being below 25%. These closely related pairs of viral genes were all from amoeba infecting mimiviruses. We selected pairs of orthologs between genes from *Escherichia coli* K-12 MG1655 and genes from other bacteria (shown in blue dots) based on the following criteria: Ka>0.01, Ks<10.0, and the percent standard error of ω being below 25%. ω values were significantly lower for Megaviridae than for bacterial homologs, indicating a higher level of evolutionary constraint on Megaviridae homologs.

The functional constraint on a protein sequence may be estimated by the ratio (ω) of non-synonymous (Ka) and synonymous (Ks) substitution rates. A small ω value indicates an elevated level of functional constraint (*i.e.*, slow pace of amino acid sequence evolution), while a large ω value, which is typically smaller than 1 for a functional protein coding sequence, indicates a low level of functional constraint (*i.e.*, fast amino acid sequence evolution). We computed ω values for Megaviridae Rpb2/Rpb1 by comparing close homologs. We also computed ω values for bacterial Rpb2/Rpb1 by comparing genes of *Escherichia coli* K-12 MG1655 with those of other closely related bacteria (Fig. 1). The average ω value for Megaviridae Rpb2 was 0.0205, while it was 0.0582 for bacterial Rpb2. The average ω value for Megaviridae Rpb1 was 0.0105, while it was 0.0811 for bacterial Rpb1. These results suggest that functional constraints on Megaviridae Rpb2/Rpb1 were higher than those on bacterial homologs (*P*=0.00003 for Rpb2, *P*=0.003 for Rpb1); however, the sample sizes for Megaviridae were small (*n*=7 for Rpb2, *n*=3 for Rpb1).

### Reference trees and taxonomic classification of metagenomic sequences

**Fig. 2:**
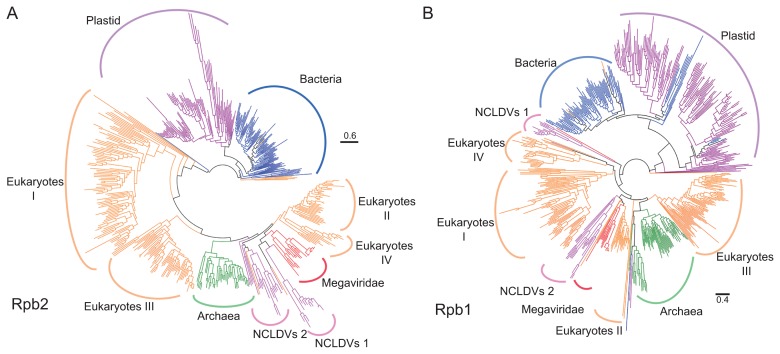
Maximum likelihood phylogenetic trees of Rpb2 and Rpb1. The Rpb2 tree (RTree-Rpb2) was constructed using 511 representative sequences (A), and the Rpb1 tree (RTree-Rpb1) with 575 representative sequences (B). Branches are colored as follows: Eukaryotes I-IV (orange), Bacteria (blue), Archaea (green), Megaviridae (red), plastid (purple), and other NCLDVs (pink).

We identified 59,938 Rpb2 and 40,534 Rpb1 homologs in the UniProt/RefSeq sequence databases using profile HMMs derived from COG0085 (Rpb2) and COG0086 (Rpb1). Viral Rpb2/Rpb1 sequences identified by this search all originated from NCLDVs. Among these sequences, 511 Rpb2 and 575 Rpb1 sequences were selected as reference sequences after reducing redundancy by clustering and discarding unusually long and short sequences. Based on the reference sequences, we built reference phylogenetic trees for Rpb2 (Fig. 2A) and Rpb1 (Fig. 2B). The reference trees were generally consistent with the classification of prokaryotes and viruses as well as eukaryotic paralogs.

Using profile HMMs built from these reference sequences, 248,101 and 252,609 sequences were obtained from metagenomes as candidates of environmental Rpb2 and Rpb1, respectively. These environmental sequences were phylogenetically classified using the reference trees described above, and specific phylogenetic groups were successfully assigned to 195,195 Rpb2 and 214,521 Rpb1 sequences (Table 1). The taxonomic assignments were dominated by Bacteria (80% for Rpb2, 81% for Rpb1), Archaea (5.7% for Rpb2, 6.6% for Rpb1), and Megaviridae (10.2% for Rpb2, 6.1% for Rpb1) as expected from the microbial size fractions (enriched with prokaryotic size organisms and viruses, Table S1) targeted by most of the analyzed metagenomes. Most of the sequences that were taxonomically assigned to Megaviridae were found in marine metagenomes (Rpb2: 18,633 [93.3%]; Rpb1: 12,225 [93.0%]), which is consistent with the previous finding of the high abundance of Megaviridae in the sea (32). The detection of eukaryotic sequences was limited (1,824 for Rpb2 and 1,276 for Rpb1 for RNA polymerase II from marine metagenomes) and likely biased towards picoeukaryotes due to the filter size range. Therefore, we excluded eukaryotic sequences in subsequent analyses and focused on Megaviridae, Bacteria, and Archaea sequences identified in marine metagenomic data unless otherwise specified.

### Taxon richness of Megaviridae RNAP is greater than those of Bacteria and Archaea

**Fig. 3:**
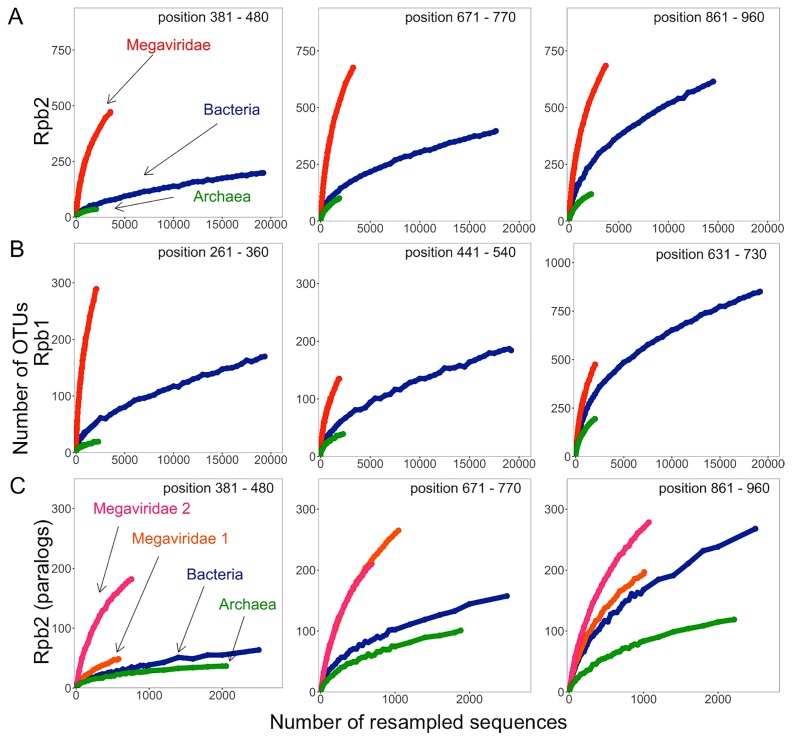
Rarefaction curves of richness for metagenomic Rpb2/Rpb1 sequences. The X-axis indicates the numbers of resampled sequences for each organism group and the Y-axis indicates the average number of OTUs over 10 resamplings. Sequence clustering was performed using an 80% amino acid sequence identity cut-off. Regarding each Rpb2 (A, C) and Rpb1 (B), three positions of the reference alignment were selected for comparisons of taxon richness between Megaviridae (red), Bacteria (blue), and Archaea (green). In (C), the taxon richness of two paralogous groups of Megaviridae Rpb2 (pink/orange) were assessed separately.

**Fig. 4:**
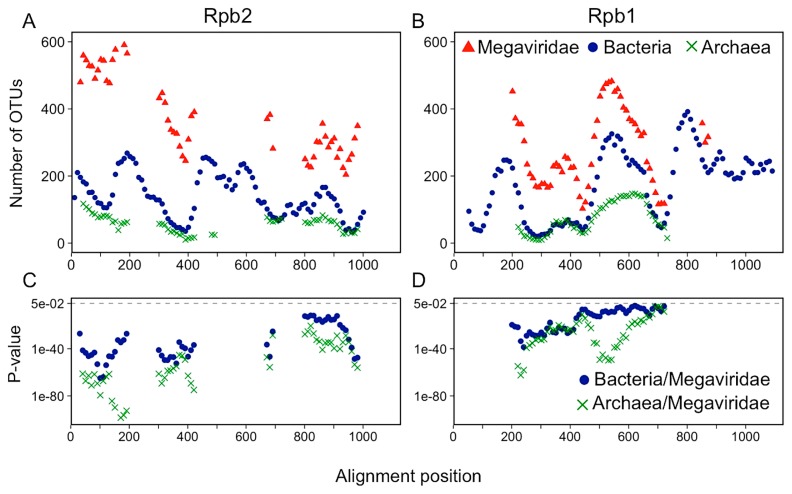
Richness for 1,000 metagenomic Rpb2/Rpb1 sequences along the length of reference alignments. The numbers of OTUs after the resampling of 1,000 metagenomic sequences were plotted at each sequence region of Rpb2 (A) and Rpb1 (B). The significance (p-value) of differences between Megaviridae and prokaryotes was assessed using the Log-rank test at each sequence region of Rpb2 (C) and Rpb1 (D).

The average lengths of the Rpb2 and Rpb1 reference sequences were as follows: Megaviridae Rpb2 (1,239 aa) and Rpb1 (1,392 aa); bacterial Rpb2 (1,282 aa) and Rpb1 (1,346 aa); archaeal Rpb2 (1,132 aa) and Rpb1 (1,373 aa). In contrast, most of the metagenomic Rpb2/Rpb1 sequences were found to be partial: Megaviridae Rpb2 (314 aa) and Rpb1 (314 aa); bacterial Rpb2 (292 aa) and Rpb1 (285 aa); archaeal Rpb2 (313 aa) and Rpb1 (303 aa). These sequences were aligned on reference Rpb2 and Rpb1 alignments (RAln-MBA-Rpb2 and RAln-MBA-Rpb1) composed of complete sequences from Megaviridae, Bacteria, and Archaea (Fig. S1). We assessed taxon richness by generating operational taxonomic units (OTUs) from sequences aligned inside a 100-aa window along the reference alignments. In order to generate OTUs, clustering was performed with three amino acid sequence identity thresholds (*i.e.*, 90%, 80%, and 70% identities). Rpb2 and Rpb1 of Megaviridae showed a higher number of OTUs than those of Bacteria or Archaea at all resampling levels at each of the three arbitrarily selected sequence rich regions (Fig. 3 and Fig. S2A and S2B). Similar results were obtained when metagenomic sequences from other environments, such as freshwater and the human gastrointestinal tract, were included (Fig. S2C and S2D), and confirmed along the entire length of the reference alignments; Megaviridae exhibited a larger number of OTUs than the two cellular domains whatever the regions of Rpb2 and Rpb1 considered (Fig. 4A and B). Log-rank tests indicated that differences in the number of OTUs between Megaviridae and the two cellular domains were significant (Fig. 4C and D).

### Effects of the existence of Megaviridae Rpb2 paralogs

Rpb2 and Rpb1 were encoded as a single copy in most of the sequenced bacterial and archaeal genomes; only 1.96% (Rpb2) and 2.97% (Rpb1) of bacterial, and 1.00% (Rpb2) and 1.00% (Rpb1) of archaeal genomes presented paralogs. However, during the reconstruction of RNAP reference trees, we noted that some Megaviridae, such as PgV and OLPV1/2, encoded two copies of Rpb2 genes. The existence of these paralogs may contribute to increasing the richness of the homologous group of sequences, hence inducing bias in taxon richness interpretations. In order to investigate the evolutionary relationships of these paralogs, we reconstructed Rpb2 trees, including metagenomic sequences, based on the same three sequence rich sub-alignment regions. The results of these analyses revealed that the Rpb2 paralogs were only distantly related in the reconstructed phylogenetic trees (Fig. S3). A set of Rpb2 from PgV, OLPV1, and OLPV2 grouped together, whereas another set of Rpb2 from the same viruses formed another group. This tree topology strongly suggested a single duplication event of Rpb2 in the ancestor of these viruses. Therefore, the existence of Megaviridae Rpb2 paralogs may lead to an approximately two-fold increase in apparent richness. In order to obtain a more reasonable estimate for the taxon richness of Megaviridae based on Rpb2 sequences, we classified metagenomic Rpb2 homologs into two groups by taking putative ancient duplication into account (Fig. S3A, S3B, and S3C). Richness estimates and rarefaction curves for individually analyzed paralogous groups still indicated a larger number of OTUs for Megaviridae than for Bacteria and Archaea at any given number of resampled sequences (Fig. 3C). Paralogs were not found for Rpb1, except for a pair of Rpb1 sequences in AaV. Sequence identity between the AaV Rpb1 sequences was 33%. These sequences were found to be closely located in phylogenetic trees when metagenomic Rpb1 sequences were included (Fig. S3D). Therefore, we considered the influence of the paralogous Rpb1 groups on the taxon richness estimate to be negligible.

### Comparison of PD between Megaviridae and Bacteria/Archaea

**Fig. 5:**
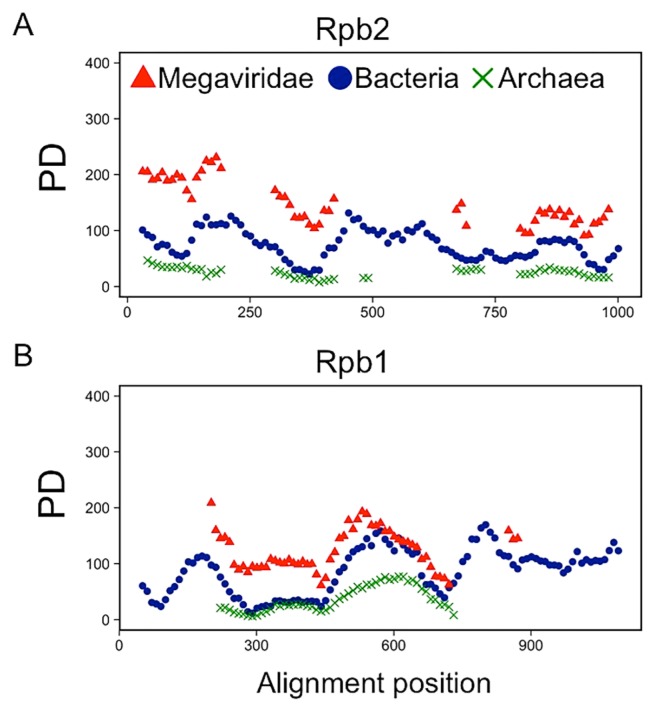
Phylogenetic diversity of metagenomic Rpb2/Rpb1 sequences along the length of reference alignments. Phylogenetic diversity (PD) scores were computed with phylogenetic trees constructed using 1,000 metagenomic sequences at each sequence region of Rpb2 (A) and Rpb1 (B).

PD is a measure of the diversity of phylogenetically related sequences, defined as the sum of all branch lengths in the phylogenetic tree (60). We calculated PD scores for Rpb2 and Rpb1 sequences obtained from marine metagenomes using a sliding window on the Rpb alignment RAln-MBA-Rpb2 and RAln-MBA-Rpb1 (Fig. 5). Megaviridae showed higher PD scores than Bacteria and Archaea along the entire length of the alignments.

**Fig. 6:**
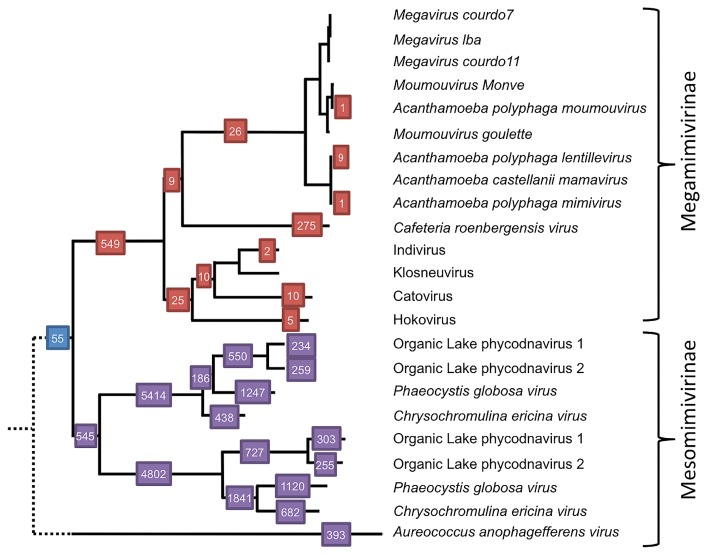
Numbers of metagenomic sequences assigned to branches of Megaviridae Rbp2. This phylogenetic tree is part of the full phylogenetic tree in Fig. 2A. The numbers in red squares are the numbers of sequences in the Megamimivirinae subfamily and those in purple are the numbers of the Mesomimivirinae subfamily. The dashed branch lines near the root of the tree represent the status of AaV sequence not forming a monophyletic group with the other members of Megaviridae in the full reference tree in Fig. 2A.

The phylogenetic distribution of Megaviridae Rpb2 sequences on the reference tree indicated that a larger number of metagenomic sequences mapped to Mesomimivirinae subfamily branches (95.1%) than to Megamimivirinae subfamily branches (4.6%) (Fig. 6). Among the Megamimivirinae branches, a larger number of environmental sequences (275 sequences) mapped to the branch leading to CroV than to the branches leading to Klosneuviruses (52 sequences) or amoebal mimiviruses (37 sequences). A notable feature of the Rpb2 phylogenetic distribution was that the deeper the branches (*i.e.*, the closer to the root), the higher the number of environmental sequences they got assigned: *e.g.* 5,414 sequences mapped to the root of one of the Mesomimivirinae Rpb2, whereas only 234, 259, 1,247, and 438 sequences mapped to the leaves representing OLPV1, OLPV2, PgV, and CeV reference genomes, respectively.

## Discussion

In the present study, we extracted environmental Rpb1 and Rpb2 sequence fragments from a large set of microbial metagenomes (58 projects) and classified them into taxonomic groups using a phylogenetic placement method. Taxonomic assignments revealed a large representation of bacterial Rpb1/2 sequences (~80%) and fewer archaeal and Megaviridae sequences (5–10%). Megaviridae sequences were preferentially detected from metagenomes originating from aquatic environments. This is consistent with previous findings, although members of Megaviridae have been isolated from various environments including oceans, lakes, rivers, air conditioning cooling systems, drainage, and soil (41). The over-representation of bacterial Rpb1/2 sequences in metagenomes is expected given their known dominance in various environments (26, 32). When the same sequence similarity thresholds were applied for taxon delineation for cellular organisms and viruses, Megaviridae showed significantly higher taxon richness than Bacteria and Archaea. As a more general measure that does not require sequence identity thresholds, we also examined PD. The PD indices of Megaviridae were also systematically higher than those of Bacteria and Archaea.

A possible reason for why apparent taxon richness in the Megaviridae is so vast could be a fast evolutionary rate in Megaviridae. However, our results indicated that functional constraints are higher for Megaviridae Rpb1/2 than for bacterial homologs. This result suggests that the rate of sequence evolution is lower for Megaviridae Rpb1/2 than for bacterial homologs if their mutation rates are similar. Blanc-Mathieu and Ogata (5) previously indicated that the mutation rate of giant viruses may be as low as prokaryotes based on Drake’s rule, postulating that “the mutation rate per genome has evolved towards a nearly invariant value across taxa”, as well as the finding that giant viruses encode many DNA repair enzymes. A recent study monitoring more than one year of experimental evolution consistently demonstrated that the mutation rates of a giant virus, *Lausannevirus*, and a bacterium remained similar over the length of the experiment (58). Therefore, the average mutation rate of Megaviridae may be similar to that of prokaryotes.

Even if a high mutation rate potentially contributed to accelerated evolution, fast evolution is not sufficient to explain the prominent radiation of evolutionarily deep lineages because radiation requires niche expansion (62). As a matter of fact, the richness of prokaryotes, which evolve faster than eukaryotes, is less than that of eukaryotes in marine environments. Recent studies revealed the presence of ~110,000 OTUs at the species level for eukaryotic plankton in the global sunlit ocean (18), but only 36,000–45,000 OTUs for prokaryotes in the same type of environment (76, 92). The markedly high taxon richness of Megaviridae revealed by our study parallels the high richness of eukaryotes, the potential hosts of Megaviridae. Based on the ancient origin of Megaviridae that has been inferred to antedate the emergence of major eukaryotic lineages, our results strongly support the Megaviridae family having a phylogenetically deep and wide co-evolutionary history with diverse marine protists. This virus-host co-evolution may have been at work from the early “Big-Bang” radiation down to the more recent diversification of the tree of eukaryotes. In other words, the long history of the diversification of eukaryotes may have played a key role in the successive niche expansion of Megaviridae. A similar co-evolutionary history was also proposed for a family of RNA viruses (37).

Many of the Megaviridae sequences were placed in the branches leading to Mesomimivirinae (Fig. 6), which are currently represented by algae-infecting viruses, such as PgV and CeV. The host range of algal species of this clade spans from Haptophyceae (Phaeocystales and Prymnesiales) to Pelagophyceae and Chlorophyta, which are deeply separated from one another in the eukaryotic tree. It is also important to note that even haptophytes alone constitute a very rich group of unicellular eukaryotes (18). Among the Megamimivirinae subfamily, one of the most abundant lineages observed in marine metagenomes was the microzooplankton infecting CroV; however, since the deeper branches also received many sequence assignments, the inference of potential hosts for these sequences are difficult. Overall, the phylogenetic positions of these marine Megaviridae marker genes point to diverse protists, including unicellular algae and microflagellates, as the potential host of these uncultured Megaviridae. Although the amoebal co-culture method (41) has permitted many new mimiviruses to be analyzed, further efforts to isolate viruses from diverse eukaryotes are desirable in order to increase the genome sampling coverage of this diverse clade.

In the present study, we showed that the taxon richness of Megaviridae exceeded that of the prokaryotic domains in the ocean. Investigations on the as yet uncovered diversity of Megaviridae will require the development of experimental alternatives to virus isolation by co-culture method, which is a labor-intensive process depending on the culturability of eukaryotic hosts. These methods include single cell genomics (87), single virus genomics (84), the development of degenerate PCR primers, and a co-occurrence network analysis (32).

## Supplementary Material



## Figures and Tables

**Table 1:** Number of taxonomically assigned metagenome sequences.

	Environment									
	
	Marine		Other aquatic		Mammal associated		Other		Total	
					
Operational clade name	Rpb2	Rpb1	Rpb2	Rpb1	Rpb2	Rpb1	Rpb2	Rpb1	Rpb2	Rpb1
Eukaryote I	690	741	16	14	82	109	0	2	788	866
Eukaryote II	1,824	1,276	77	28	78	108	4	2	1,983	1,414
Eukaryote III	729	854	17	10	68	101	0	1	814	966
Eukaryote IV/V	82	54	7	5	0	3	0	0	89	62
Bacteria	111,124	125,874	6,387	6,740	38,192	39,798	588	625	156,291	173,037
Archaea	10,177	12,826	640	784	56	102	300	341	11,173	14,053
Megaviridae	18,633	12,225	1,330	841	10	79	0	0	19,973	13,145
Chloroplast	2,540	7,666	27	455	91	1,159	2	5	2,660	9,285
NCLDVs 1	119	80	20	6	19	9	0	0	158	95
NCLDVs 2	1,135	1,484	126	110	4	2	1	2	1,266	1,598
Total	147,053	163,080	8,647	8,993	38,600	41,470	895	978	195,195	214,521
