# Supplementary material for: Taxon Richness of “Megaviridae” Exceeds those of Bacteria and Archaea in the Ocean
Source: Microbes Environ. 2018 May 25;33(2):162–71. doi: 10.1264/jsme2.ME17203 (PMC6031395; doi:10.1264/jsme2.ME17203)
Supplement: Supplementary file 1 [file 33_162_s1.pdf]

Table S1. Metagenomic data used in this study.

MARINE	Filter Min-Max	No of ORFs	No of Rpb2	No. of Rpb1
**Total**		**101,856,227**	**147,053**	**163,080**
Tara Oceans[a]	-0.22, 0.1-0.22, 0.22-0.B1245, 0.45-0.8, 0.2-1.6 or 0.2-3.0 µm	61,655,613	90,507	99,453
Tara Oceans (454)[b]	0.22-1.6 µm	1,421,586	2,810	3,398
CAM_PROJ_GOS	0.002-0.22, 0.1-0.8, 0.22-0.8, 0.8-3 or 3-20 µm	11,301,013	15,664	17,783
CAM_P_0000692	0.2-20 µm	7,092,526	13,349	14,458
CAM_PROJ_AntarcticaAquatic	0.1-, 0,1-0.8, 0.8-, 0.8-3, 3- or 3-200 µm	7,748,903	11,138	12,163
CAM_P_0001109	0.1-0.8, 0.8-3 or 3-200 µm	3,360,214	5,516	5,926
CAM_PROJ_BotanyBay	0-3, 0.1-0.8, 0.8-3 or 1-1 µm	4,276,800	2,858	4,060
CAM_P_0001069	0.1-, 0.8- or 3.0- µm (-20 µm)	1,986,898	2,020	2,166
CAM_P_0001026	0.22-5 µm	506,581	933	1,100
CAM_P_0000712	0.2- µm	266,981	453	539
CAM_PROJ_WesternChannelOMM	0.22- µm	251,564	260	403
CAM_P_0000545	0.2- µm	106,660	159	208
CAM_PROJ_EpibiontMetagenome	NA	146,343	262	257
CAM_PROJ_AlvinellaPompejana	NA	65,605	239	231
CAM_P_0000828	-0.22 µm	95,931	135	157
CAM_PROJ_HOT	0.22-1.6 µm	120,944	115	119
CAM_PROJ_WhaleFall	NA	89,035	101	118
CAM_P_0001133	0.1-, 0.8- or 3.0- µm	90,028	122	145
CAM_PROJ_GutlessWorm	NA	197,680	110	105
CAM_PROJ_HydrothermalVent	NA	43,384	58	55
CAM_PROJ_HypersalineMat	NA	161,473	80	74
CAM_P_0001129	0.2-5 µm	4,379	12	13
CAM_P_0001028	0.8-3 µm	29,319	21	34
CAM_P_0001196	*	28,426	21	32
CAM_P_00001027	NA	181,508	26	23
CAM_P_0000912	-1 µm	153,798	24	18
CAM_P_0000915	0.22- µm	254,039	24	15
CAM_P_0000914	0.2-2.7 µm	194,763	10	10
CAM_PROJ_DeepMed	0.22-5 µm	6,181	11	2
CAM_PROJ_PacificOcean	0.2-5 µm	5,623	7	8
CAM_PROJ_PBSM	NA	12,429	8	7
**OTHER AQUATIC ENVIRONEMENT**				
**Total**		**8,385,210**	**8,647**	**8,993**
environmental_T30141[c]	NA	2,412	0	0
CAM_PROJ_YLake	0.8- and 3- µm	3,139,742	4,135	3,955
CAM_P_0001136	NA	2,568,652	1,311	1,343
CAM_P_0001174	0.1-0.8, 0.8-3 or 3-200 µm	739,134	1,508	1,720
CAM_PROJ_BisonMetagenome	NA	679,211	666	669
CAM_P_0001130	NA	216,114	327	404
CAM_P_0001131	NA	188,446	201	315
CAM_PROJ_WashingtonLake	NA	471,326	137	142
CAM_P_0001132	0.1-, 0.8- or 3.0- µm	205,930	188	193
CAM_P_0001128	0.2-5 µm	98,421	112	167
CAM_PROJ_ViralSpring	-0.2 µm	18,943	28	29
CAM_PROJ_Yellowstone	NA	56,879	34	56
**MAMMAL ASSOCIATED**				
**Total**		**38,341,510**	**38,600**	**41,470**
organismal[c]	NA	37,169,008	37,617	40,461
CAM_PROJ_HumanGut	100-100 µm	670,835	439	471
CAM_PROJ_HumanDistalGut	NA	219,736	260	219
CAM_PROJ_TwinStudy	NA	126,420	181	188
CAM_P_0000523	NA	42,367	86	112
CAM_PROJ_MouseGut	NA	15,204	13	9
CAM_P_0000909	-0.45 µm	97,940	4	10
**OTHERS**				
**Total**		**1,063,049**	**895**	**978**
environmental_T30140[c]	NA	8,675	0	0
CAM_PROJ_AcidMine	0.45- or 0.2- µm	229,511	489	492
CAM_PROJ_EBPRSludge	NA	198,391	158	134
CAM_PROJ_TermiteGut	NA	79,498	120	155
CAM_PROJ_SAM	0.2- µm	394,096	41	89
CAM_PROJ_FarmSoil	NA	113,301	43	56
CAM_P_0000504	-1000 µm	32,745	37	46
CAM_P_0000911	NA	6,832	7	6

[a] Sunagawa et al., Science, 348, 1261359 (2015).
[b] Hingamp et al., ISME J, 7, 1678-1695 (2013).
[c] KEGG/MGENES (http://www.genome.jp/mgenes/).
* Metadata were not available.
NA Filter size data were not available in the metadata.

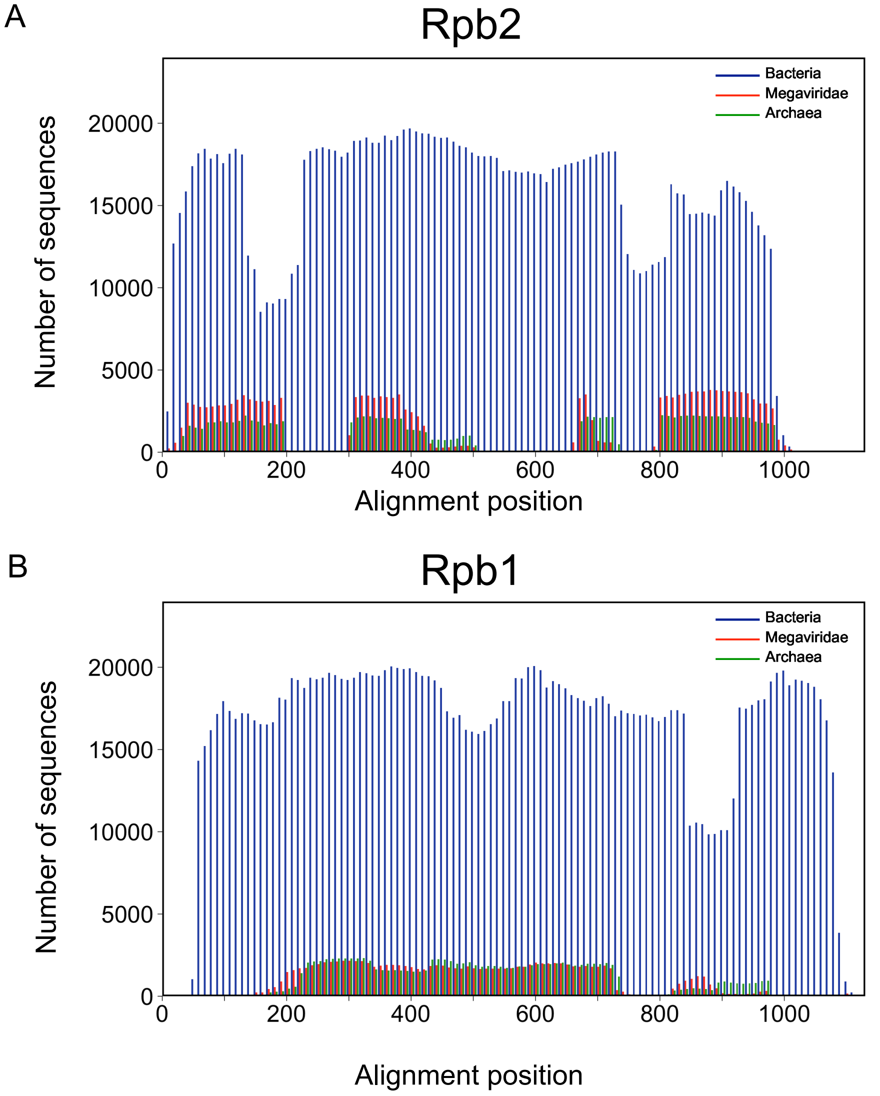

Supplementary Fig. S1. Distribution of metagenomic sequences mapped on the reference alignments. (A) Metagenomic sequences mapped on the Megaviridae, Bacteria and Archaea Rpb2 reference alignment (RAln-MBA-Rpb2). (B) Metagenomic sequences mapped on the Megaviridae, Bacteria and Archaea Rpb1 reference alignment (RAln-MBA-Rpb1). Metagenomic sequences analyzed here were derived from marine metagenomes. X-axis indicates positions in the reference alignments and Y-axis represents the number of sequences. Color codes are red for Megaviridae, blue for Bacteria and green for Archaea.

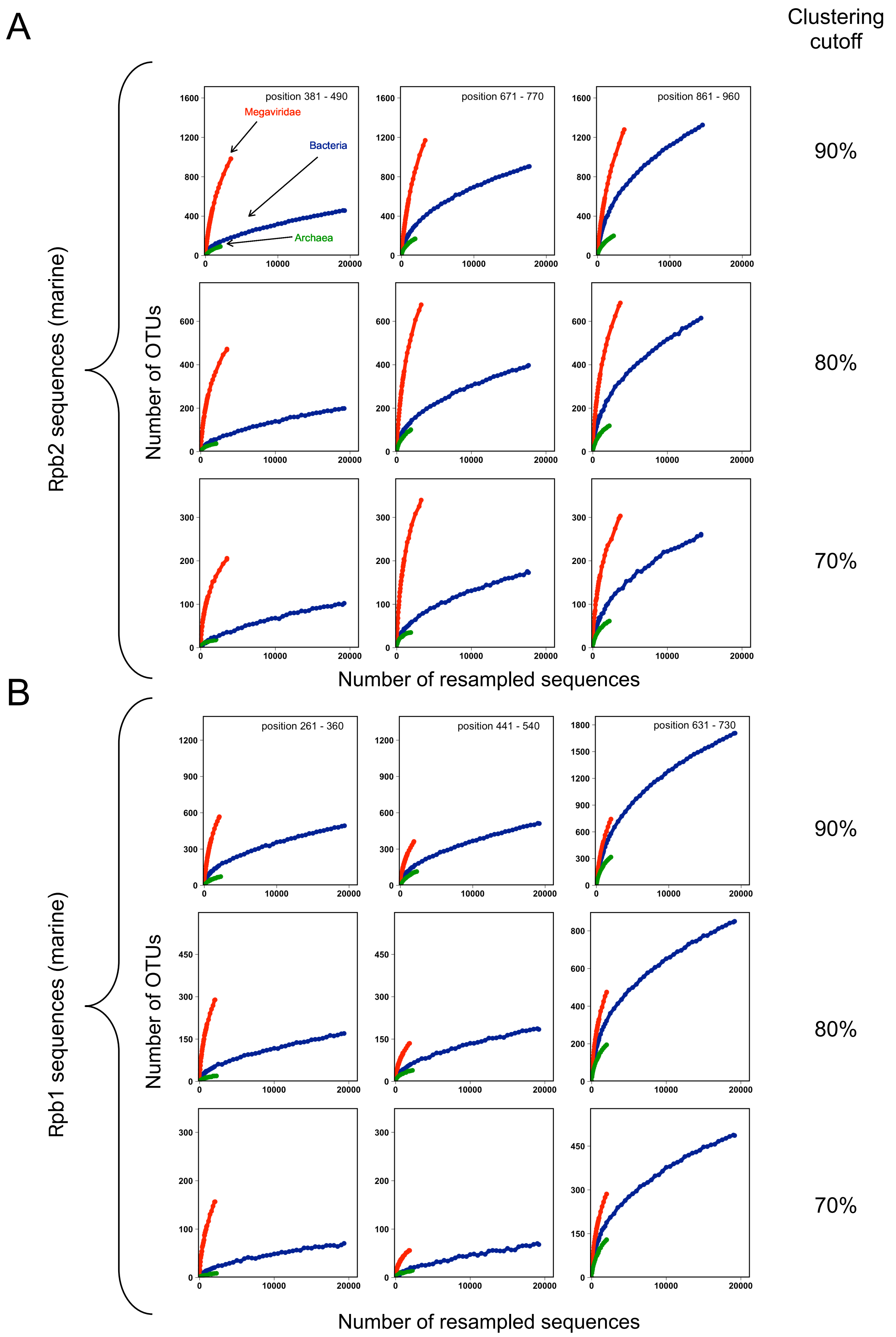

Supplementary Fig. S2. Rarefaction curves of richness for metagenomic Rpb2/Rpb1 sequences. Three cutoff values (90%, 80% and 70%) were used to generate OTUs. (A) Rarefaction curves for marine metagenomic Rpb2 sequences. (B) Rarefaction curves for marine metagenomic Rpb1 sequences. (C) Rarefaction curves for total metagenomic Rpb2 sequences. (D) Rarefaction curves for total metagenomic Rpb1 sequences.

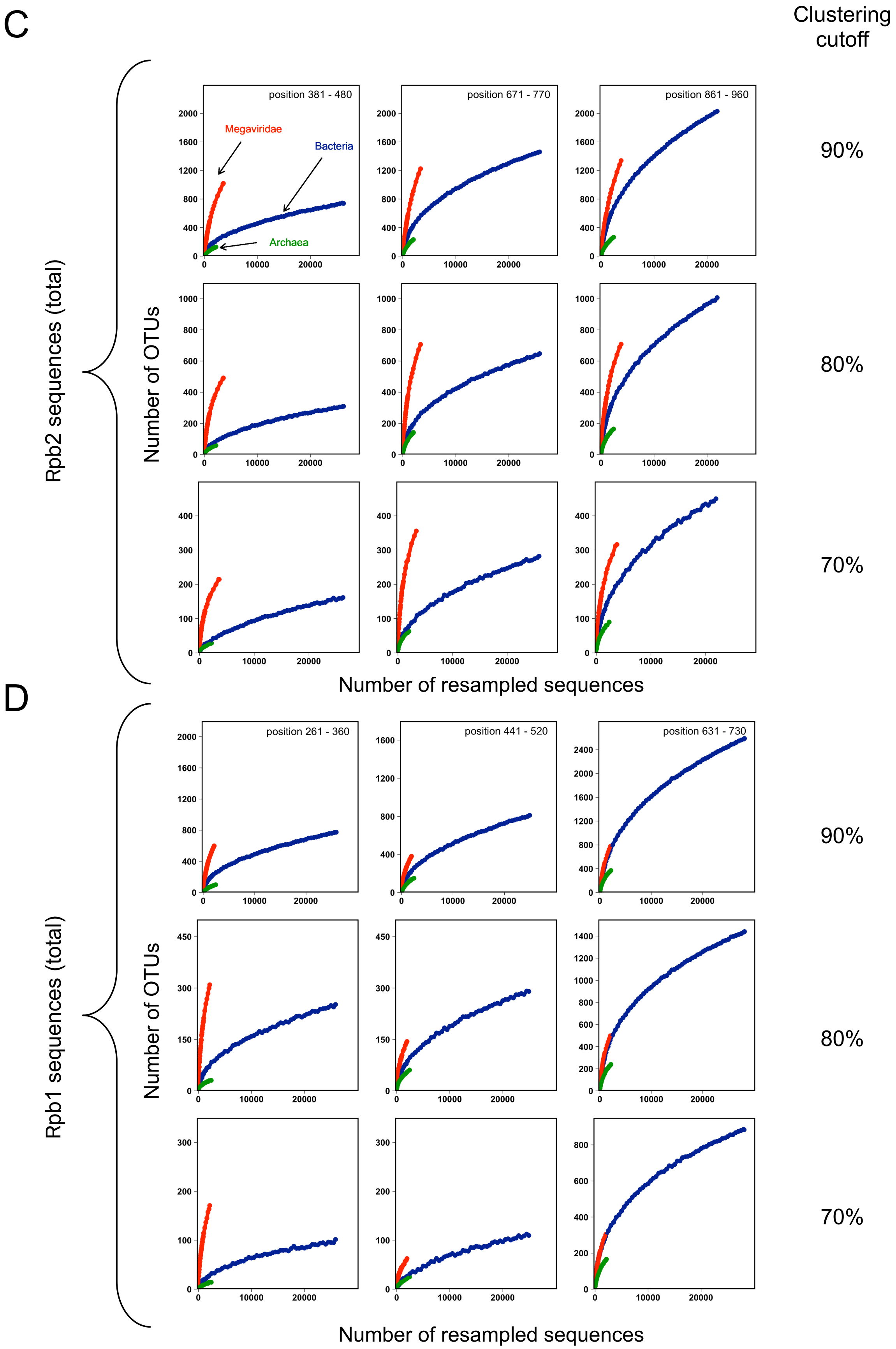

Supplementary Fig. S2 (Continued)

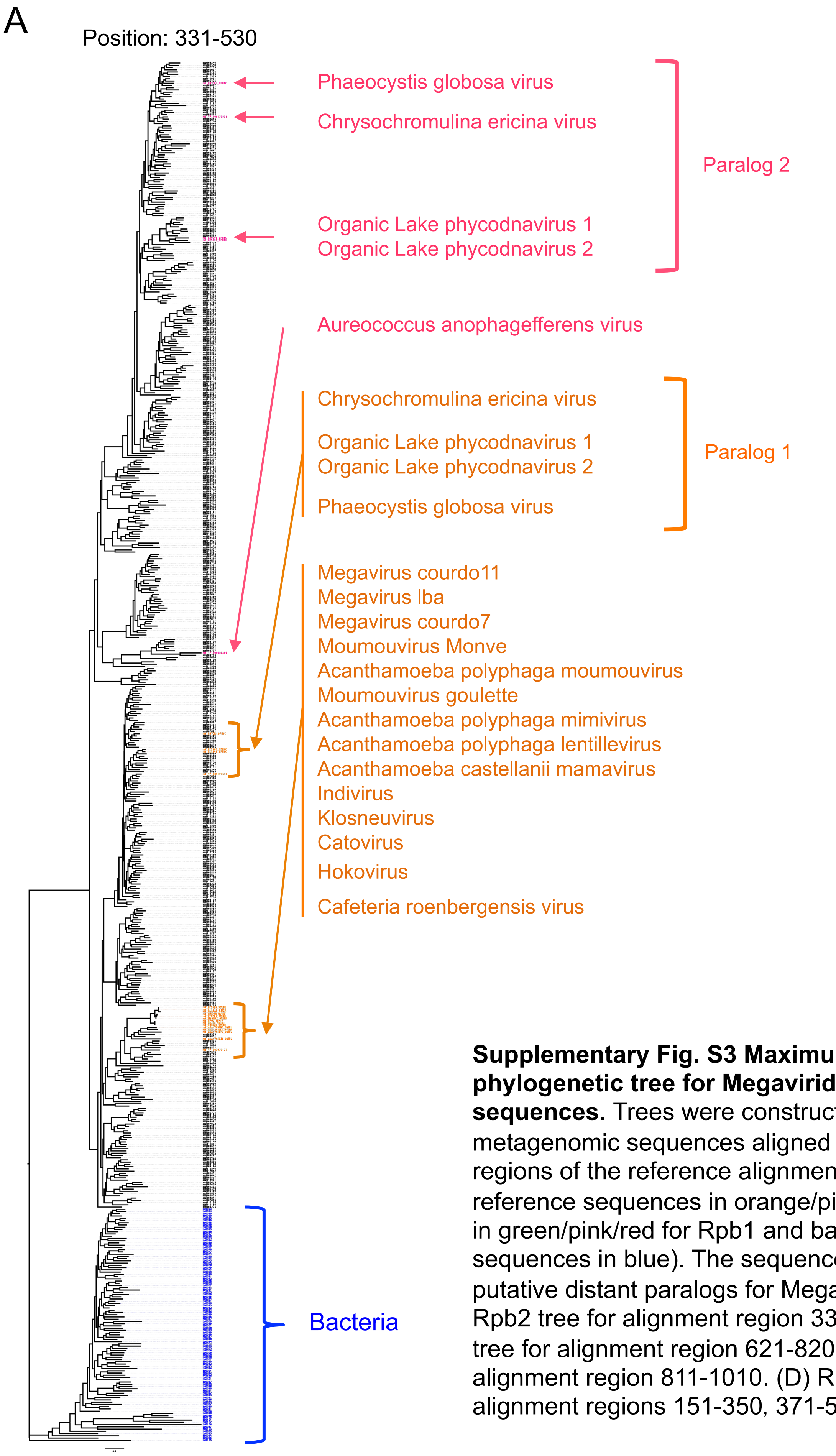

Supplementary Fig. S3 Maximum likelihood phylogenetic tree for Megaviridae metagenomic sequences. Trees were constructed from marine metagenomic sequences aligned on distinct regions of the reference alignment (Megaviridae reference sequences in orange/pink for Rpb2 and in green/pink/red for Rpb1 and bacterial reference sequences in blue). The sequences in pink indicate putative distant paralogs for Megaviridae Rpb2. (A) Rpb2 tree for alignment region 331-530. (B) Rpb2 tree for alignment region 621-820. (C) Rpb2 tree for alignment region 811-1010. (D) Rpb1 trees for alignment regions 151-350, 371-570, and 551-750.

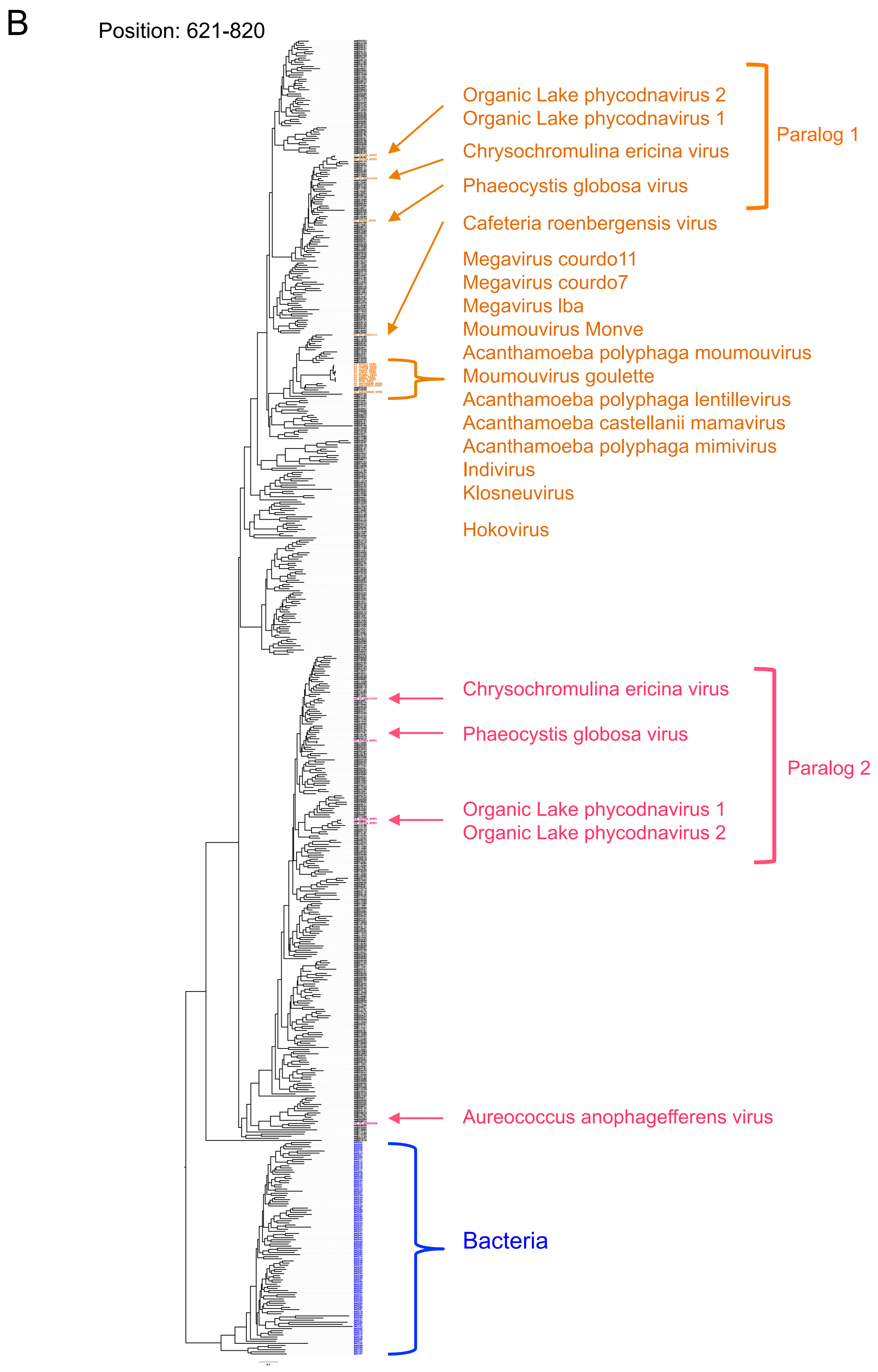

Supplementary Fig. S3 (Continued)

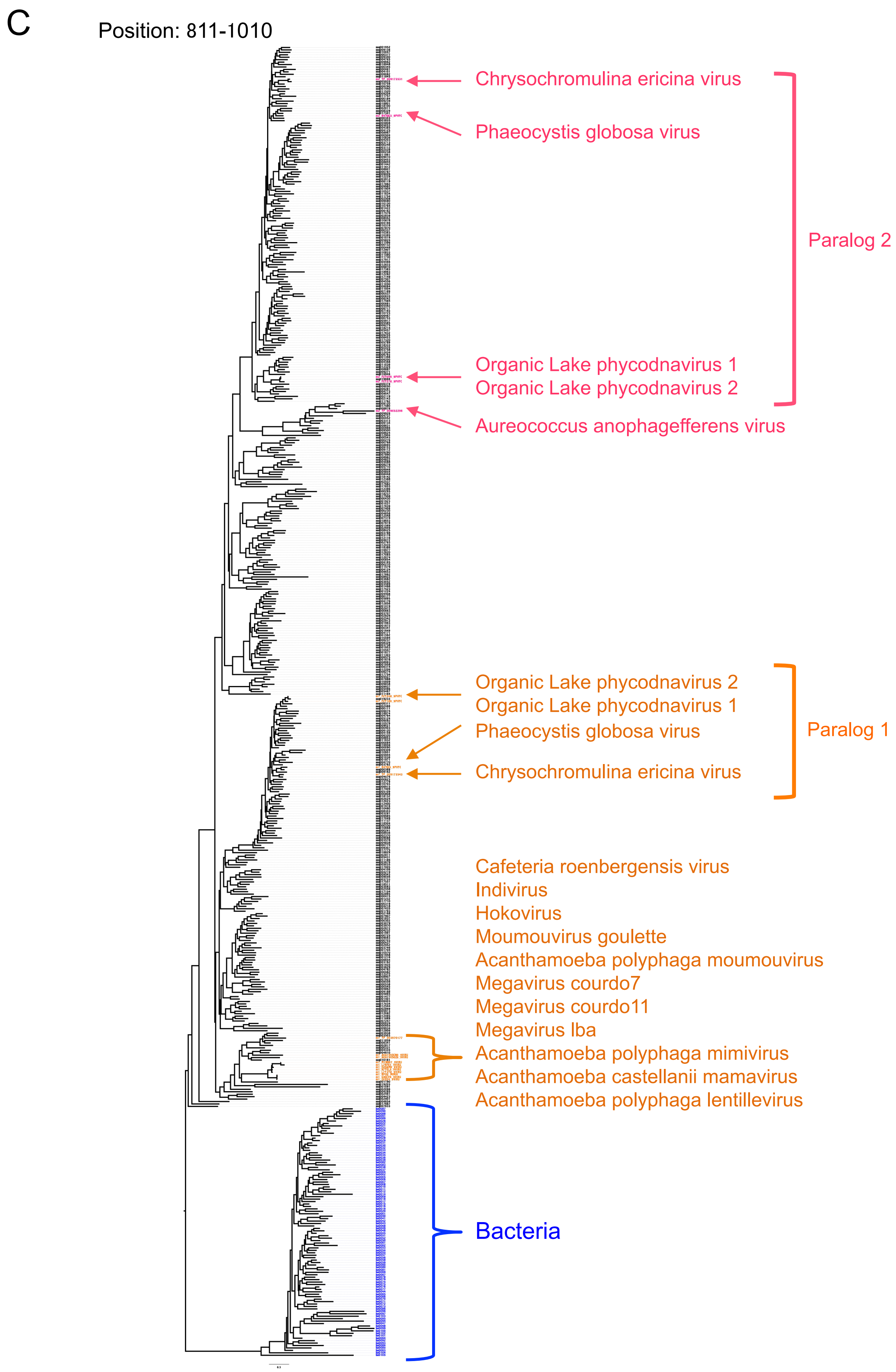

Supplementary Fig. S3 (Continued)

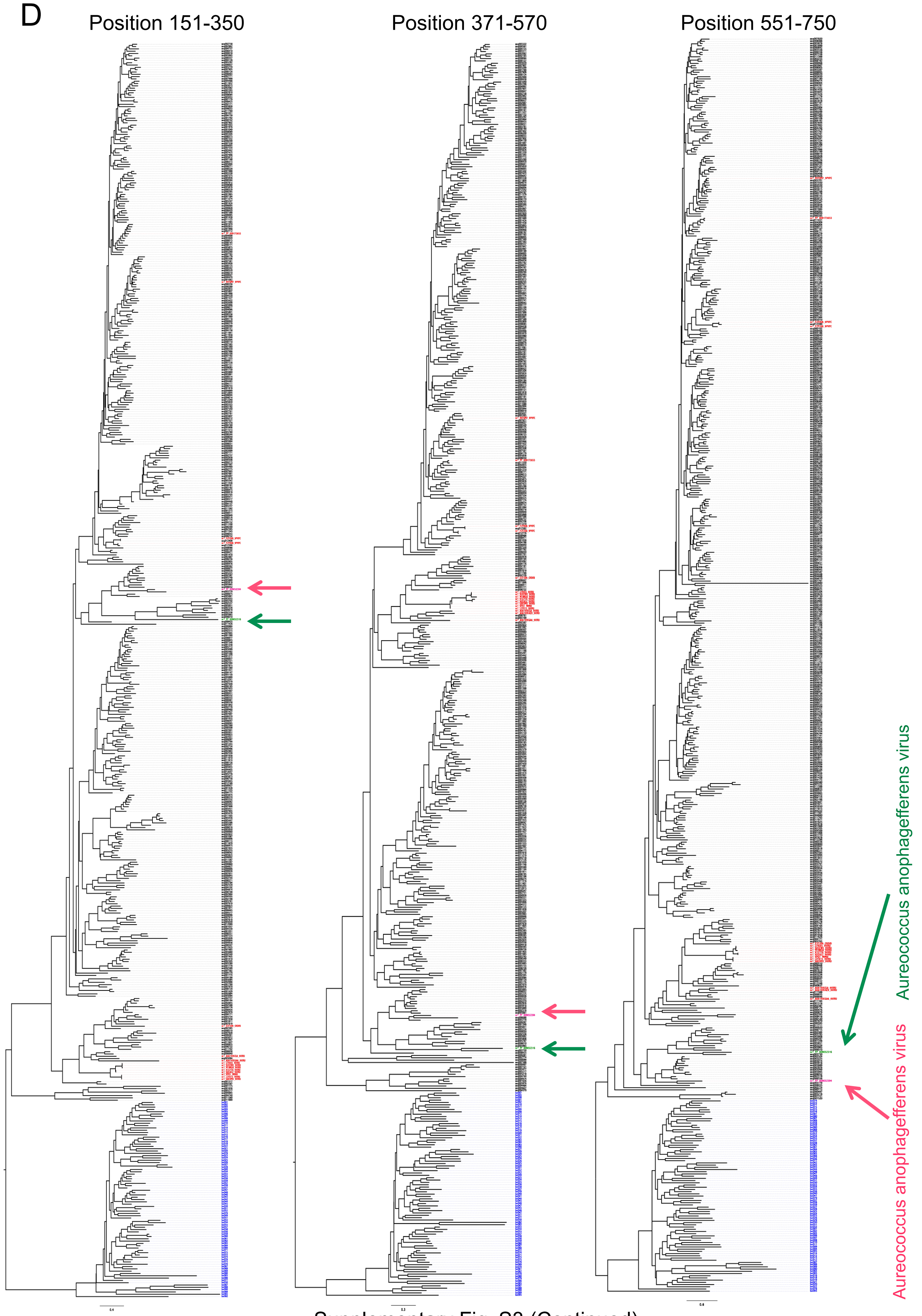

Supplementary Fig. S3 (Continued)
